# Audit on availability, quality and frequency of clincal and educational supervision

**DOI:** 10.1192/bjo.2021.249

**Published:** 2021-06-18

**Authors:** Gayathri Gnanasekaram, Amanda Hoar

**Affiliations:** Somerset partnership NHS trust

## Abstract

**Aims:**

GMC defines clinical supervisor as a trainer who is responsible for overseeing a specified trainee's clinical work throughout a placement in a clinical or medical environment and is appropriately trained to do so¹.

This AUDIT aimed to review the frequency, content and quality of clinical supervision for psychiatric trainees within Somerset NHS Foundation Trust. Both Severn deanery and Somerset NHS Foundation Trust both recommend psychiatry trainees have one hour of supervision per week, involving exploration of trainee clinical and educational needs.

**Method:**

All trainees working in Somerset NHS Foundation Trust psychiatry from February 2020 were invited to participate. A survey was designed to quantify the frequency of supervision amongst this cohort. Survey online software, SurveyMonkey, was chosen for the accessibility and user friendly modality and disseminated via email to all junior doctors (n = 27). Survey responses were collected in the last month of the placement (July–August 2020).

Questions on accomplishing workplace based assessments (WPBA), managing e-portfolio requirements were asked, with Likert scale responses available. Quality of supervision was explored via white space answers.

Surveys were reviewed by the AUDIT authors and descriptive data collected.

**Result:**

63% trainees responded (17 out of 27). Educational objectives were discussed at the beginning of the placement. Over half the respondents stated that time was not set aside to look at e-portfolio.

Workplace based assessments (WBPAs), and Case based discussions (CBDs) were more frequently achieved than observed assessments of clinical encounters (ACEs/Mini-ACEs) (assessment of clinical encounter).

30% core psychiatry trainees respondents (4 out of 7) discussed their audits/QI projects with their supervisors most/always. 42% (3 out of 7) had a discussion sometimes.

2 GP and foundation trainees stated they were unable to obtain community mental health experience. The response rate to this question was disappointing and we think it may be secondary to the pressures of the pandemic.

100% respondents described educational supervisors as supportive and approachable.

**Conclusion:**

Whilst all respondents found their supervisors approachable and supportive, completion of formal WPBAs and portfolio reviews was suboptimal.

Following regional presentation of results, the pertinence of these findings for all trainees was highlighted. A supervision template has been created and extension of this initial audit to a regional quality improvement project is underway.

Specific recommendations included brief and regular supervisor check-ins with trainees regarding projects and psychotherapy competencies and a mid-placement review of portfolio.

